# Does the postoperative quality of reduction, regardless of the surgical method used in treating a calcaneal fracture, influence patients’ functional outcomes?

**DOI:** 10.1186/s12891-023-06697-z

**Published:** 2023-07-10

**Authors:** Sayyed-Hadi Sayyed-Hosseinian, Matin Shirazinia, Hamid Arabi, Monavar Afzal Aghaee, Ehsan Vahedi, Farshid Bagheri

**Affiliations:** 1 Orthopedic Research Center, Shahid Kamyab Hospital, Mashhad University of Medical Sciences, Mashhad, Iran; 2 Faculty of Medicine, Mashhad University of Medical Sciences, Mashhad, Iran; 3 Social Determinants of Health Research Center, Mashhad University of Medical Sciences, Mashhad, Iran; 4 Department of Orthopedic Surgery, Shahid Kamyab Hospital, Fadayian Eslam Street, Mashhad, Iran

**Keywords:** Calcaneal fractures, Sinus tarsi approach, Extensile lateral approach, Anatomic reduction

## Abstract

**Background:**

The extensile lateral approach (ELA) and sinus tarsi approach (STA) are commonly utilized for surgically treating calcaneal fractures. This study compared the outcomes of ELA and STA in the management of calcaneal fractures and assessed the influence of postoperative quality of reduction on functional and pain scores.

**Methods:**

The study included 68 adults with Sanders type-II and type-III calcaneal fractures who underwent either ELA or STA surgery. Pre- and postoperative radiographs and computed tomography scans were analyzed, and functional and pain scores were evaluated using the Manchester Oxford Foot Questionnaire (MOXFQ), American Orthopedic Foot and Ankle Society (AOFAS) ankle-hindfoot score, and Visual Analogue Score (VAS) during follow-up visits.

**Results:**

Out of the total patients, 50 underwent ELA surgery while 18 underwent STA surgery. The anatomic (excellent) reduction was achieved in 33 (48.5%) patients. There were no significant differences between the ELA and STA groups concerning functional scores, pain scores, the proportion of excellent reduction, and complications. Additionally, anatomic reduction, compared to near or non-anatomic (good, fair, or poor) reduction, demonstrated a decrease in MOXFQ (unstandardized β coefficient: -13.83, 95% CI: -25.47 to -2.19, p = 0.021), an increase in AOFAS (unstandardized β coefficient: 8.35, 95% CI: 0.31 to 16.38, p = 0.042), and a reduction in VAS pain (unstandardized β coefficient: -0.89, 95% CI: -1.93 to -0.16, p = 0.095) scores.

**Conclusion:**

In conclusion, we found no significant differences regarding complications, excellent reduction, and functional scores between STA and ELA surgeries. Therefore, STA may be an effective alternative for the treatment of calcaneal fractures in Sanders type II and type III calcaneal fractures. Furthermore, the anatomic reduction of the posterior facet correlated with improved functional scores, emphasizing the importance of achieving it for restoring foot function regardless of surgery type or time between injury and surgery.

## Background

Calcaneal fractures are the most frequent fractures among all tarsal injuries, accounting for 1–2% of all human fractures [1, 2]. It typically results from a high-energy injury such as a fall from a height, or a motor vehicle accident [2]. As the calcaneus is the largest bone in the foot and plays a crucial role in walking and standing; calcaneus fractures have significant consequences and complications that may have a negative impact on a person’s ability to walk and perform daily activities [3, 4]. These complications include chronic pain, decreased mobility and range of motion, and heel deformity [5]. In some cases, patients may develop arthritis in the adjacent joints, leading to chronic pain and stiffness [6]. Rehabilitation, physical therapy, and surgery may be necessary to address these complications and restore the patient’s mobility and overall foot function [5].

In recent years, numerous studies have investigated various treatment options and outcomes of surgical and non-surgical approaches for calcaneus fractures. Although there is no universally accepted treatment protocol for all calcaneal fractures, experts generally agree that the optimal approach should be customized to the specific fracture pattern, the extent of soft tissue damage, the willingness of the patient to comply with the treatment plan, and underlying health conditions [7–9]. It is generally advised to opt for non-operative treatment in cases of intra-articular fractures that are non-displaced or only minimally displaced, as well as in extra-articular fractures that do not lead to significant hindfoot deformity [8]. Non-surgical treatment is also considered appropriate when surgery is not recommended due to specific contraindications [7].

As hindfoot deformities can result from untreated displaced extra-articular calcaneal fractures, surgical interventions are recommended to achieve optimal outcomes and restore function [7]. Additionally, surgical therapy lowers the risk of acquiring posttraumatic arthritis [10]. Two surgical methods, namely the extensile lateral approach (ELA) and sinus tarsi approach (STA), are frequently employed to manage fractures of the calcaneus [11]. However, controversy remains regarding which technique offers the best outcomes [6, 12, 13]. The ELA is frequently utilized and provides superior visualization of the fracture site along with precise reduction and fixation capabilities. This may lead to a reduction in the incidence of posttraumatic arthritis. Nevertheless, ELA is associated with a relatively high risk of wound dehiscence and infection [7]. In selected cases, the STA may be a reasonable alternative, as it offers the benefits of reduced soft tissue injuries [14].

Achieving anatomic reduction is essential in the treatment of calcaneal fractures, given the calcaneus’ intricate structure, comprising several articular surfaces, which may result in severe consequences if not handled properly [6, 15]. Due to the controversies regarding the best method of surgery, we designed this study to compare the outcomes of ELA versus STA in the surgical management of calcaneal fractures. Additionally, we evaluated the impact of postoperative anatomic reduction on the foot functional outcomes and pain score.

## Methods

### Study population

This retrospective cohort study was conducted from March 2018 to March 2021. The list of all patients who had a prior history of calcaneal fracture and were admitted to our hospital, were obtained. The hospital that took part in the study serve as the main referral center for traumatic patients in Mashhad, Iran.

Inclusion criteria were adult patients (older than 18 years), close fractures, and fractures managed surgically using the STA or ELA. To restrict the effect of the type of fractures on our results, we only chose the Sanders type-II and type-III fractures. Younger patients, open calcaneal fractures, and fractures that had been treated conservatively or surgically with approaches other than ELA and STA were excluded. All surgeries were done by a single surgeon.

Afterward, all patients received an invitation with details regarding the study’s purpose. Individuals who declined to participate or did not reply to the invitation were also excluded from the study.

### Surgical approaches

#### Extensile lateral approach (ELA)

The procedure was carried out by positioning the patient in a lateral decubitus position, utilizing a beanbag on a translucent table, while under general or spinal anesthesia and with a thigh tourniquet. To start, the lower extremity was exsanguinated, and then access to the calcaneus was obtained through an L-shaped incision. This incision commenced laterally, approximately 3–4 cm above the calcaneal tuberosity and 1–2 cm in front of the heel cord. The incision was lengthened in a downward direction and extended toward the back of the fibula until it reached the point where the dorsal and plantar skin meet. At this junction, a gentle curve was created, directing the incision towards the front, specifically targeting the calcaneocuboid joint and the base of the fifth metatarsal. A thick subperiosteal flap was created to protect the soft tissues. The fracture line located at the Gissane angle was detected, and the lateral wall was moved downward to reveal the fractured articular parts. The depressed articular fragments were elevated using a small periosteal elevator and temporarily fixed to the constant fragment with K-wires. The focus shifted towards restoring the proper dimensions of the calcaneus by achieving full alignment of the fracture fragments. During the surgery, the surgeon ensured the alignment of the joint by assessing the reduction from various angles using C-arm imaging. The lateral view, axial view, and Broden view were examined for this purpose. Subsequently, a 4.0 mm lag screw was inserted from the outer cortex towards the sustentaculum. For stabilization of the calcaneus, a calcaneal locking plate was utilized. This plate served to stabilize the anterior process, posterior facet, and posterior tuberosity of the calcaneus. Once a secure fixation was obtained, the wound was closed in multiple layers.

#### Sinus tarsi approach (STA)

The surgical approach was conducted with the patient positioned in a lateral decubitus position on a translucent table, using a beanbag for support. General or spinal anesthesia was administered, and a thigh tourniquet was utilized. A straight incision was made on the lateral side of the foot, positioned just below the tip of the fibula and approximately parallel to the sole of the foot. The incision started about 1 cm posterior to the fibula and extended distally for around 3–4 cm. The joint surface was exposed, and the peroneal tendons retracted to gain access to the lateral wall. Then, initially, the surgeon disimpacted and reduced the tuberosity fragment, relieving it from varus deformity using a percutaneous Schanz pin. Subsequently, an axial view was taken, and K-wires were inserted into the calcaneus or talus to maintain the reduction of the tuberosity. Afterward, the surgeon proceeded to address the reduction of the posterior facet under visualization, mobilizing the fracture fragment with a small elevator. Provisional fixation was performed from lateral to medial, and the articular reduction was verified using fluoroscopy, examining lateral, axial, and Broden’s views. Simultaneously, the height and length of the calcaneus, as well as any remaining varus deformity, were corrected. Subsequently, a 4.0 mm lag screw was inserted across the fracture site in a lateral-to-medial direction, providing stability to the sustentacular bone. The posterior side of the calcaneus was accessed for percutaneous placement of 6.5 mm cannulated screws. In most cases, a low-profile plate was then placed to stabilize the anterior to posterior main fragments. Once successful fixation was achieved, the incision sites were closed layer by layer.

### Measurements

#### Measurement during the admission

Patient medical records were reviewed to gather demographic information, details on the trauma mechanism, comorbidities, and surgical methods. Additionally, the hospital’s picture archiving and communication system (PACS) was used to evaluate pre- and postoperative radiographs and computed tomography (CT) scans. The intra articular fractures were classified according to Sanders classification [16]. Moreover, patients’ imaging was used to measure Gissane and Bohler’s angle in pre and post operation. The preoperative CT scan was assessed to determine fracture fragments and displacement. The patients underwent another CT scan early post-operation and prior to discharge. The quality of reduction of the articular surface of posterior facet was assessed based on findings of postoperative CT scan. We classified the quality of reduction into four categories: excellent (anatomic), good, fair, and poor, based on previously published work [17]. All imaging interpretation were evaluated by a single foot and ankle surgeon.

#### Post operation management and measurement during the follow-up visits

All the patients were visited two weeks later. Any signs of wound complication were recorded and managed properly. Physical therapy was started as soon as the wound recovered, and sutures were removed. Ankle radiographs were taken 6 and 12 weeks after the surgery to check the union of the fragments.

On the final follow up visit, standard ankle radiographs were taken to assess Gissane and Bohler’s angle, the presence of osteoarthritis in adjacent joints, as well as non-union and malunion. The presence of osteoarthritis was identified based on Kellgren-Lawrence classification [18]. The patients’ functional capacity was evaluated using the validated Manchester Oxford Foot Questionnaire (MOXFQ) and American Orthopedic Foot and Ankle Society (AOFAS) ankle-hindfoot score [19–21]. In addition, pain was assessed using Visual Analogue Score (VAS). Moreover, patients were asked about any reoperation and returning to their previous job. All data were gathered in a predesigned checklist.

### Ethical considerations

The ethical committee of the Mashhad University of Medical Sciences approved the study protocol IR.MUMS.MEDICAL.REC.1397.393.

### Statistical analysis

Stata version 13 (Stata, College, Statin, Texas) was employed for data analysis. Normality was evaluated by Shapiro-Wilk test and P-P plot. The results were presented as either mean and standard deviation (SD) or median and interquartile range (IQR: percentile 25 to percentile 75), depending on normal distribution. The comparison of Gissane and Bohler’s angle before and after the surgery were compared using either a Paired T-test or Wilcoxon test (depending on the normality of the data distribution). Univariable analyses were also used to compare ELA and STA group. For quantitative variables, non-normally distributed data was analyzed using the Mann-Whitney U-test, while normally distributed data was analyzed using the independent t-test. Fisher exact test and Chi-square test were utilized for categorical variables.

Linear regression analysis was employed to determine the impact of postoperative quality of reduction, time between injury and surgery, and type of treatment on functional scores (AOFAS, and MOXFQ) and VAS pain score. Regarding the assumption and concerns of linear regression model, we assessed normality of residuals, homoscedasticity, collinearity, leverage, and outliers. The residuals normality was determined using P-P plot. Constant variance was evaluated using *rvfplot* and *estat imtest* post estimation command and in the case of heteroscedasticity, a robust regression was used. Furthermore, the collinearity was determined by variance inflation factor (VIF). The acceptable range for studentized residuals considered as -3 to 3. Furthermore, we considered leverage to be high if it exceeded 0.18 (3*(k + 1)/n). A statistical level of p < 0.05 (2-tailed) was considered significant for all analyses.

## Results

### Baseline characteristics

**Fig. 1:**
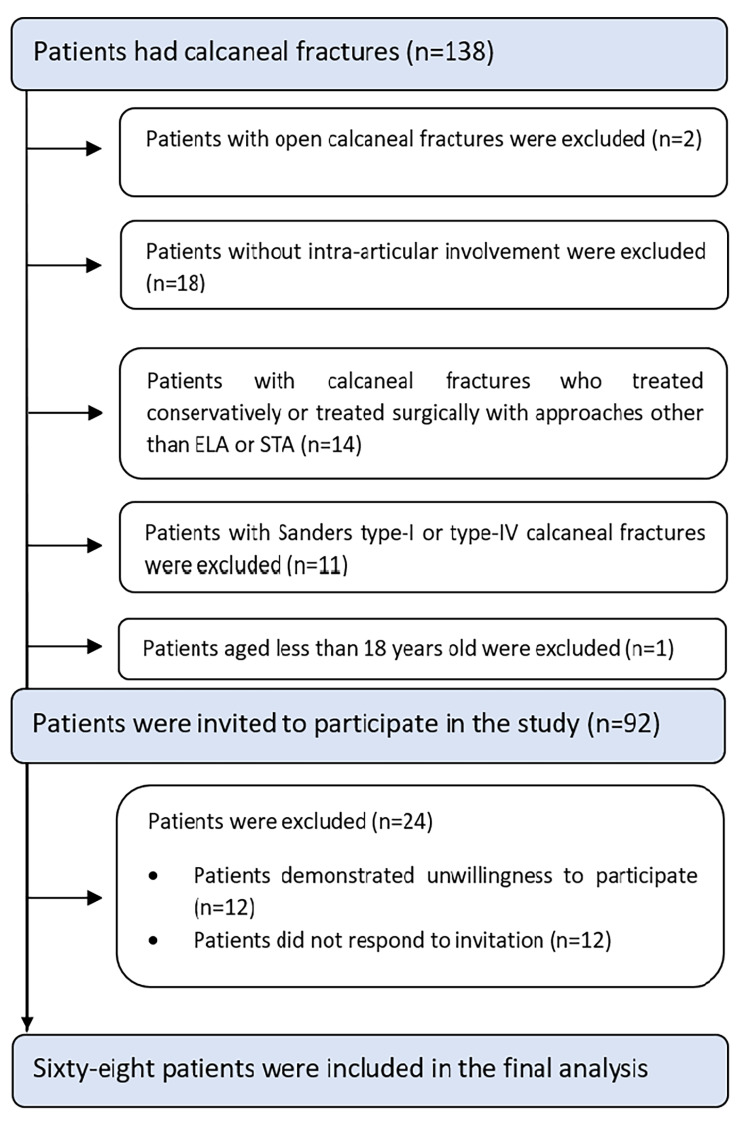
Patients flow diagram

Between March 2018 to March 2021 a total of 68 patients were included in the study of whom 58 (85.3%) were male (Table 1; Fig. 1). The mean age was 40.8 (10.9) years. The most prevalent mechanism of fracture was falling from height in 47 subjects (81.0%). Fracture of L2 was the most common concomitant fracture in 9 (13.3%) patients followed by L1 and L3 fracture each in 6 (8.8%).

**Table 1:** Characteristics of patients with calcaneus fractures

	STA (N = 18)	ELA (N = 50)
Age (years), mean (standard deviation)	42.8 (10.8)	40.1 (11.0)
Gender, male %	15 (83.3)	43 (86.0)
Comorbidities, %		
Diabetes mellitus	1 (5.6)	2 (4.0)
Cardiovascular diseases	0	3 (6.0)
Cerebrovascular Accident	1 (5.6)	1 (2.0)
Smoking, %	4 (22.2)	12 (24.0)
Opium addiction, %	4 (22.2)	4 (8.0)
Mechanism of trauma, %		
Fall from height	16 (88.9)	31 (62.0)
Motor vehicle accident	2 (11.1)	19 (38.0)
Affected limb, %		
Right	11 (61.1)	31 (62.0)
Left	7 (38.9)	19 (38.0)
Time from injury to surgery, median (percentile 25 to percentile 75)	7.0 (3.8 to 11.0)	5.0 (1.0 to 8.0)

ELA, extensile lateral approach; STA, sinus tarsi approach

The postoperative quality of reduction of articular surface was excellent reduction in 33 patients (48.5%), good in 26 (38.2%), fair in 8 (11.8%), and poor in one (Table 2). Out of all study patients, 50 were treated using ELA surgery and 18 by STA surgery.

**Table 2:** Radiologic characteristics of patients with calcaneus fractures

	STA (N = 18)	ELA (N = 50)
Type of intra-articular involvement, %		
Type 2 A	7 (38.9)	9 (18.0)
Type 2B	4 (22.2)	6 (12.0)
Type 2 C	1 (5.6)	3 (6.0)
Type 3AB	5 (27.7)	23 (46.0)
Type 3AC	1 (5.6)	7 (14.0)
Type 3BC	0	2 (4.0)
Postoperative quality of reduction, %		
Excellent	8 (44.4)	25 (50.0)
Good	10 (55.6)	16 (32.0)
Fair	0	8 (16.0)
Poor	0	1 (2.0)

ELA, extensile lateral approach; STA, sinus tarsi approach

**Fig. 2:**
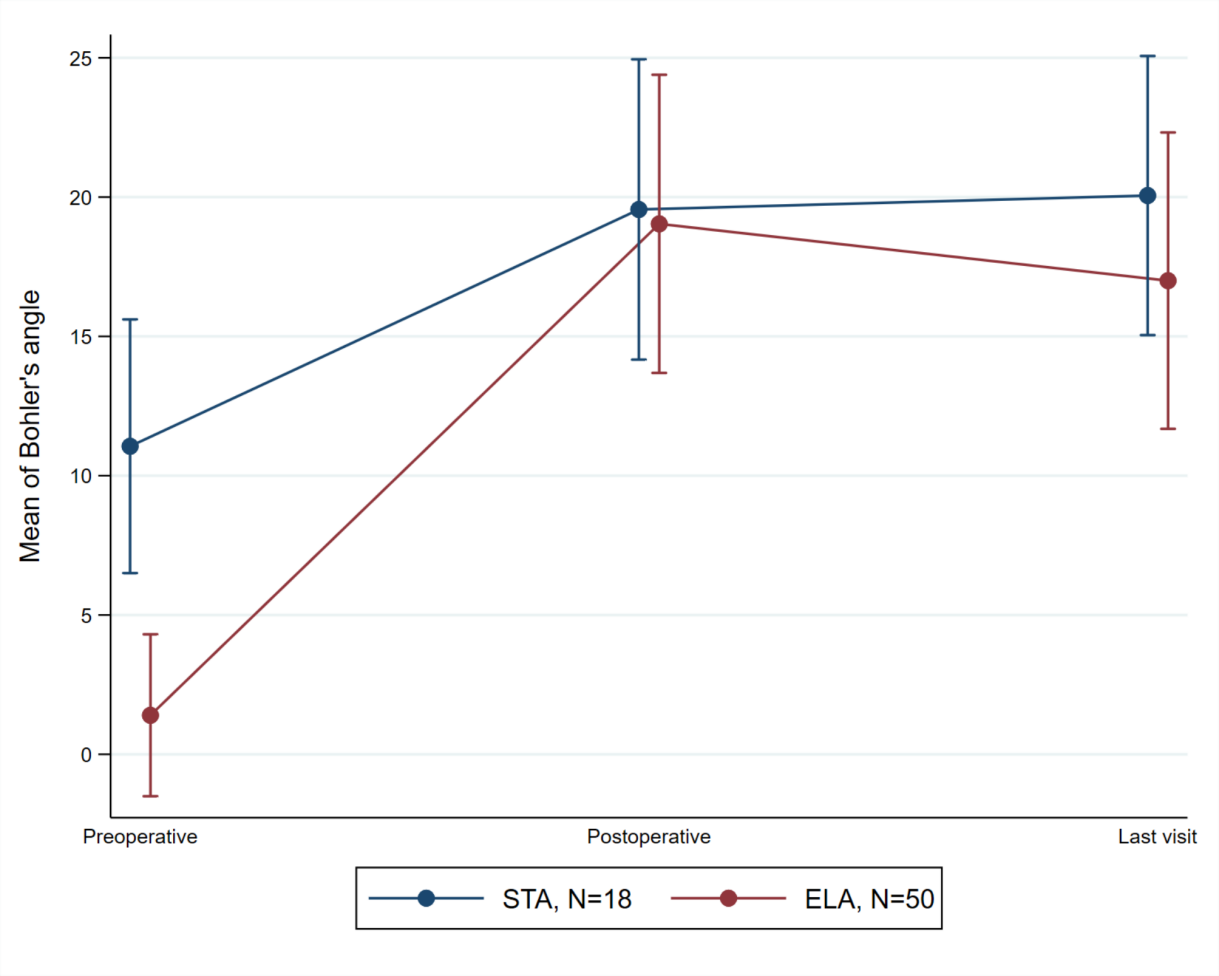
The evaluation of the Bohler’s angle in preoperative, postoperative, and follow-up visit time-points. The points and error bars indicated mean and the 95% confidence interval, respectively. ELA, extensile lateral approach; STA, sinus tarsi approach

**Fig. 3:**
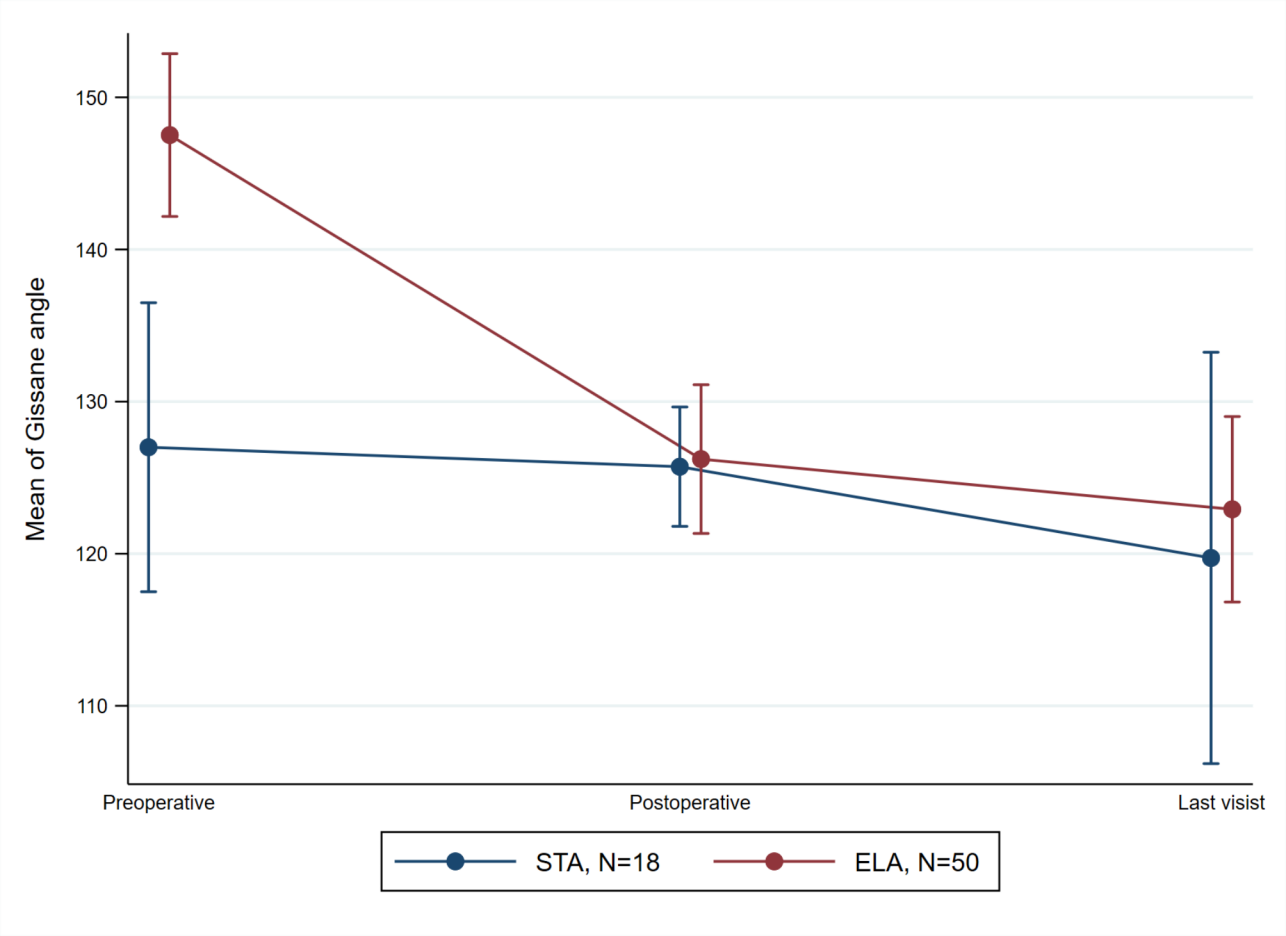
The evaluation of the Gissane angle in preoperative, postoperative, and follow-up visit time-points. The points and error bars indicated mean and the 95% confidence interval, respectively. ELA, extensile lateral approach; STA, sinus tarsi approach

After the surgery compared to before surgery, there was a significant increase in the Bohler angle in both the ELA (p < 0.001) and STA (p = 0.006) groups, as shown in Fig. 2. Also, Gissane’s angle exhibited a significant decrease in the ELA (p < 0.001) group while this was not significant for STA (p = 0.727) group, following the surgery (Fig. 3).

Wound complications including wound infections and dehiscence occurred in 6 (8.8%) patients. All of these patients were in ELA group.

### Follow-up visit

The median follow-up time was 16.5 (IQR, 11 to 32.8) months. The most frequent reported complications were osteoarthritis in 56 patients (82.4%), chronic plantar pain in 16 (23.5%), and malunion in 5 (7.4%).

Thirteen (19.1%) patients needed reoperation which the most common cause of it, was hardware removal (N = 10, 14.7%) followed by subtalar arthrodesis and debridement (N = 2, 2.9% for each). The debridement in both cases was performed during the initial admission, while both subtalar arthrodesis procedures were conducted three years after the fractures. The range for hardware removal was 3–12 months following the first operation. Forty-nine (72.1%) patients had the ability to return to their previous job.

### Comparing ELA vs. STA

The univariable analyses showed no significant difference between STA group ELA group in terms of functional and pain scores (p = 0.232 for MOXFQ score, p = 0.144 for AOFAS score, and p = 0.380 for VAS pain score) (Table 3).

**Table 3:** Univariable analysis functional and pain scores in ELA vs. STA methods

	ELA, N = 50	STA, N = 18	P-value
MOXFQ score, mean (SD)	57.2 (25.5)	49.5 (22.8)	0.232
AOFAS score, mean (SD)	72.3 (18.8)	80.3 (10.8)	0.144
VAS pain score, mean (SD)	3.68 (2.12)	3.2 (2.4)	0.380

ELA, extensile lateral approach; STA, sinus tarsi approach; SD, standard deviation; MOXFQ, Manchester-Oxford Foot Questionnaire; AOFAS, American Orthopaedic Foot and Ankle Society; VAS, visual analogue scale

The proportion of excellent reduction was not significantly different in STA group in comparison to ELA group (p = 0.786) (Table 4). Regarding complications and returning to the previous job, there were no significant differences observed between the two groups of surgeries (p > 0.05 for all comparisons).

**Table 4:** Univariable analysis of outcomes and complications in ELA vs. STA methods

	ELA, N = 50	STA, N = 18	P-value
Excellent reduction, %	25 (50.0)	8 (44.4)	0.786
Wound complications, %	6 (12.0)	0	0.184
Subtalar joint osteoarthritis, %	44 (88.0)	12 (66.7)	0.068
Calcaneocuboid joint osteoarthritis, %	14 (28.0)	2 (11.1)	0.203
Chronic plantar pain, %	14 (28.0)	2 (11.1)	0.203
Malunion, %	5 (10.0)	0	0.315
Need for reoperation, %	11 (22.0)	2 (11.1)	0.488
Return to the previous job, %	36 (72.0)	13 (72.2)	1.000

ELA, extensile lateral approach; STA, sinus tarsi approach

### Association between the different predictors and functional and pain scores

The results of the multivariable linear regression analysis showed that excellent reduction, when compared to good, fair, or poor reduction, was significantly associated with a decrease in MOXFQ (unstandardized β coefficient: -13.83, 95% CI: -25.47 to -2.19, p = 0.021) and an increase in AOFAS (unstandardized β coefficient: 8.35, 95% CI: 0.31 to 16.38, p = 0.042). Furthermore, excellent reduction compared to other quality of reduction caused a marginally significant reduction in VAS pain score (unstandardized β coefficient: -0.89, 95% CI: -1.93 to -0.16, p = 0.095) (Table 5).

Other predictors did not reach statistical significance except for the type of surgical approach in AOFAS model. STA, in comparison to ELA, showed a significant increase (unstandardized β coefficient: 8.81, 95% CI: 1.41 to 16.21, p = 0.020) in AOFAS score.

All assumptions and concerns were met in our models, except for homoscedasticity in the case of AOFAS. For the AOFAS model, we used a robust regression model.

**Table 5:** Multivariable linear regression analysis of the effect of admission predictors on functional and pain scores

Response variable	Predictors	Unstandardized β coefficient (95% CI)	Standardized β coefficient	P-value	Adjusted r^2^
MOXFQ score	The time between injury to surgery (less than two weeks^1^)	-12.13 (-29.30 to 5.05)	-0.17	0.163	0.077
	Type of surgery (STA^2^)	-9.00 (-22.19 to 4.19)	-0.16	0.178	
	Postoperative quality of reduction (excellent^3^)	-13.83 (-25.47 to -2.19)	-0.28	**0.021**	
AOFAS score^4^	The time between injury to surgery (less than two weeks^1^)	6.20 (-4.57 to 16.98)	0.12	0.255	0.112
	Type of surgery (STA^2^)	8.81 (1.41 to 16.21)	0.23	**0.020**	
	Postoperative quality of reduction (excellent^3^)	8.35 (0.31 to 16.38)	0.24	**0.042**	
VAS pain score	The time between injury to surgery (less than two weeks^1^)	-1.03 (-2.57 to 0.51)	-0.16	0.186	0.031
	Type of surgery (STA^2^)	-0.61 (-1.79 to 0.57)	-0.12	0.306	
	Postoperative quality of reduction (excellent^3^)	-0.89 (-1.93 to -0.16)	-0.21	0.095	

MOXFQ, Manchester-Oxford Foot Questionnaire; AOFAS, American Orthopaedic Foot and Ankle Society; VAS, visual analogue scale; STA, sinus tarsi approach; 95% CI, 95% confidence interval

^1^The reference category was more than two weeks

^2^The reference category was the extensile lateral approach

^3^The reference category was good, fair, or poor postoperative quality of reduction

^4^Due to heteroscedasticity, robust regression was used

## Discussion

The current study evaluated the outcomes of 68 patients with type II and III calcaneal fractures. We compared the postoperative quality of reduction, complications, and the functional and pain scores of patients who underwent ELA or STA surgery. The findings of present study showed that STA and ELA did not significantly differ in terms of functional and pain scores (MOXFQ, AOFAS, VAS pain scores), achieving anatomic (excellent) reduction, wound complications (wound infection and dehiscence), rate of subtalar and calcaneocuboid joint osteoarthritis, plantar pain, malunion, need for reoperation, and returning to the previous job.

In the conventional method of ELA, direct reduction and reinforcement of the fracture fragments are made possible. Therefore, this method is expected to be more effective in achieving anatomic reduction. Although surgeons make every effort to handle the soft tissue with care during ELA, wound healing complications are still common. The problems that arise with wound healing have led to the development of less invasive methods for fixing and reducing calcaneal fractures. To name a few, arthroscopy-assisted fixation, percutaneous fixation, and the STA are some of these approaches [12]. STA is considered to be more protective of nerves and the blood supply surrounding the incision. While a body of evidence including observational study, RCTs, and meta-analyses, evaluated these two types of surgeries with each other, there are some debates regarding complications and outcomes of each. Moreover, none of previous meta-analyses evaluated the certainty of evidence [22, 23].

Some researchers suggest that, compared to ELA method, STA lacks evident clinical benefits, particularly in restoring crucial anatomic landmarks [22, 24–26]. Furthermore, in comparison with ELA, STA has a lower level of exposure to the fracture site, that may reduce the ability to guarantee correct anatomic reduction [24, 27]. A more recent study, using examination of the extent of postoperative quality of reduction in comparison to the unaffected limb, showed that the ELA method had significantly greater calcaneal width preservation than the STA method [28]. Moreover, in the study of 83 fractures, ELA generally demonstrated better reductions in the Bohler angle and posterior facet in the imaging evaluation. However, no significant difference was found between the two approaches in Sanders type II fractures [29]. Additionally, the study of Nosewicz et al. showed that a minimally invasive STA may adequately expose even in more sophisticated calcaneal fractures (Sanders type III) for anatomical reduction. According to the aforementioned study, 64% of surgeries using the STA attained excellent and good reduction [17].

Based on our findings, while the wound complications were higher in the ELA group, the difference was not significant. This may be due to low power status of our study. The rate of wound infections in ELA group in our study was in line with most of the prior study which reported it in 5–15% of patients [30–32]. However, in some studies the rate of wound infection has been reported as high as near 30% in ELA approach [33, 34]. A recent meta-analysis revealed that the odds of getting a wound infection using STA were considerably lower (72% lower) than in ELA method [22]. Giving the soft tissue enough time to recover from swelling and disappearing blisters is a pivotal key. The time between injury to surgery, higher amount of body mass index, drug abuse, smoking, placing closed suction drain, and using ELA are some factors that are associated with wound infections [35–39].

Malunion is one of the potential complications associated with calcaneal fractures [5, 40]. Both non-surgical and surgical treatments are associated with malunion, although surgical treatment appears to have a lower incidence of this complication [41]. The rate of malunion in our study is relatively low when compared to a similar study [31]. This lower rate of malunion could arise from the high proportion of excellent and good (87%) reduced cases in the present study. Another possible reason could be that we did not include type IV fractures in our study.

In the findings of the current study STA had better functional and pain scores but the result was not significant. These results are consistent with prior research that demonstrated a nonsignificant difference between the two groups concerning VAS pain and AOFAS scores [31, 42]. On the other hand, on the study of Takasaka et al., ELA showed a better AOFAS score compared to STA [43]. In our study, after adjusting for the quality of reduction and the time between injury and surgery, STA demonstrated a significant nine-score increase in AOFAS compared to ELA. However, this association was not observed in the case of other scores. Similar to this finding, a recent meta-analysis showed that the STA method had significantly higher scores of AOFAS [22].

There is a scarcity of information regarding the impact of the quality of reduction on functional scores, regardless of the surgical method employed. This issue was assessed in the present study. In cases where surgery is deemed necessary, achieving an anatomic reduction of the joint surfaces is crucial for achieving positive functional outcomes [10, 44]. Multivariable regression analysis in our study showed that anatomic (excellent) reduction in comparison to near or non-anatomic (good, fair, or poor) reduction caused a beneficial effect on functional scores. Additionally, the time between injury to surgery did not significantly associate with any functional outcomes. Also, the point estimation of this factor showed a trivial effect. Albeit, according to the time between the injury and surgery, experts suggest that if surgery is delayed, it will become more difficult to achieve anatomical reduction. This is because, during the delay, fibrous tissue may begin to form, the soft tissues around the injury may contract, and the muscles may become tighter. These factors can make it more challenging for the surgeon to align the bones correctly, which may result in a poorer outcome [45].

Our study has several limitations. The most notable limitation of our study is the small sample size of our cohort. In addition, the two groups of patients were not equal. Both issues impact the power of our study. This is important because none of parameters between the two groups of ELA and STA were statistically significant. Moreover, it should be declared that there was a considerable difference in terms of Gissane and Bohler’s angle between ELA and STA group. Taken together, an inference of equivalency in outcomes between the two groups should not be made. Second, repeated measurements of functional scores were not addressed in our study. Thus, the trend of functional scores changes was not accessible. In addition, the follow-up period of patients was not equal. Furthermore, a considerable proportion of patients did not respond to our invitation. Thus, we did not access the functional outcomes of these patients. Last but not least, all of surgeries in our center were carried out by an experienced foot surgeon, which caused less generalizability of our findings. Thus, we recommend that future studies compare the outcomes of calcaneal fracture using STA techniques in patients who were operated on by foot surgeons with those who were operated on by general orthopedic surgeons.

## Conclusion

In conclusion, STA surgery when compared to the ELA surgery did not show any significant difference in terms of complications, achieving anatomic reduction, and functional and pain scores. Therefore, based on our results, STA surgeries may be an effective alternative to ELA surgeries in Sanders type II and type III calcaneal fractures. Nonetheless, considering the limitations of our studies, further studies with prospective design and larger sample sizes need to confirm these findings.

Also, we demonstrated a significant correlation between achieving an anatomic reduction of the posterior facet’s articular surface and improved functional scores. This correlation was independent of the surgical technique employed or the duration between injury and surgery, highlighting the significance of attaining an anatomic reduction for optimal outcomes.

## Data Availability

The data underlying this article will be shared upon reasonable request to the corresponding author.

## List of abbreviations

STA: Sinus Tarsi Approach
ELA: Extensile Lateral Approach
PACS: Picture Archiving and Communication System
AOFAS: American Orthopedic Foot and Ankle Society
MOXFQ: Manchester Oxford Foot Questionnaire
VAS: Visual Analogue Score
CI: Confidence Interval
SD: Standard Deviation
IQR: Interquartile Range
VIF: Variance Inflation Factor
